# Channel-Directed Enzymatic Depolymerization within a Metal–Organic Framework

**DOI:** 10.1021/acsami.5c04137

**Published:** 2025-05-01

**Authors:** Jana Glatz, Jesús Cases Díaz, Jorge Salinas-Uber, David Talens-Perales, Julio Polaina, Mónica Giménez-Marqués

**Affiliations:** † 201469 Universidad de Valencia - Instituto de Ciencia Molecular, Catedrático José Beltrán Martínez 2, 46980 Paterna, Spain; ‡ Instituto de Agroquímica y Tecnología de Alimentos (CSIC), Avda. Catedrático Agustín Escardino 7, 46980 Paterna, Spain

**Keywords:** metal–organic-frameworks, in situ encapsulation, biocomposite, biocatalysis, enzymatic depolymerization

## Abstract

Controlled growth of metal–organic frameworks (MOFs) under mild conditions has enabled the production of hybrid biocomposites with potential applications in biocatalysis. While the structure and bioactivity of confined enzymes are retained, improving the mass transport across the porous architecture remains a challenge. Here, we report a biocompatible and scalable synthetic procedure of a phase-pure aluminum trimesate porous framework, MIL-110­(Al), featuring accessible microporous channels. The method is compatible with the *in situ* encapsulation of enzymes via a Lewis acid-mediated mineralization, reaching high efficiencies, and with control over protein loading. Moreover, we demonstrate a favored channel-directed depolymerization in a model biocomposite, xylanase@MIL-110­(Al), which successfully hydrolyzes the xylan polymer over consecutive cycles. This work emphasizes the possibility of improving the overall enzymatic performance in depolymerization reactions by using MOF-protective scaffolds featuring large accessible porosity.

## Graphical abstract

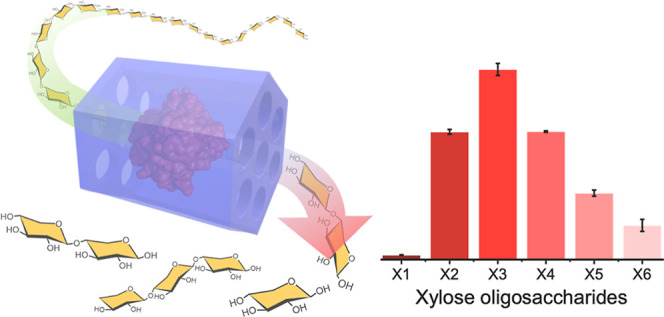

## 1 Introduction

Enzymatic depolymerization is a promising, sustainable method for breaking down complex polymers, which is particularly relevant in the current context of waste management and recycling. Compared with chemical treatment, enzymatic catalysis is a clean and efficient method. However, the structural instability of enzymes under industrial-scale conditions—elevated temperature, pH, and the presence of inhibitors—generally compromises their activity, whereas their nonrecoverable nature remains a significant barrier that hampers their broad industrialization. These hurdles can be alleviated by exploiting enzyme immobilization methods, which improve structural fragility and endow reusability performance, therefore lowering operational costs and paving the way for their ample use. Porous reticular materials including metal–organic frameworks (MOFs),^1–3^ covalent–organic frameworks (COFs),^4,5^ and hydrogen-bonded organic frameworks (HOFs)^6,7^ have emerged as promising scaffolds for assembling bioentities, primarily through surface immobilization or encapsulation. When encased within reticular matrices, enzymes preserve structural integrity and retain their bioactivity due to the matrices’ high surface areas and tailorable pore sizes, which facilitate substrate diffusion and adapt substrate local concentrations. The composition and architecture of porous scaffolds also play a crucial role in controlling cargo release,^3,8–10^ enabling a gating effect that enhances biocatalytic selectivity^11^ and even promoting non-native biofunctionality.^12^ However, when applied to the degradation of large particle sized materials, such as polymeric substrates, these matrices often act as diffusion barriers, limiting access to the active sites. Therefore, improving the reactivity in biocatalytic depolymerizations requires the development of new scaffold architectures with larger pore apertures and expanded diffusion channels to facilitate substrate accessibility. The use of channeled mass transport within reticular materials is an effective strategy to govern biocatalysis that has primarily been explored through infiltration, particularly in mesoporous COFs and MOFs.^13^ However, its applications via *de novo* synthesis remain largely unexplored due to the challenge of reaching pore-channeled architectures under mild conditions.

Bioentity@MOF direct synthesis—the so-called coprecipitation or *in situ* mineralization^2,14,15^ offers distinct advantages, as it overcomes the limitations of infiltration methods—the need for precise pore window/enzyme size matching—while preventing translocation and preserving maximal MOF porosity.^16^ Herein, the key challenge lies in leveraging the chemical versatility of MOF materials to precisely tailor their composition and architecture for specific functions such as enhanced thermostability, facilitated mass transfer, or host-induced bioactivity. Essentially, the selection of MOF scaffolds that meet *in situ* requirements remains limited,^17^ with most studies focusing on zeolitic imidazolate framework (ZIF)-based biocomposites—particularly ZIF-8 ^2^ and isoreticular structures, ZIF-90 and MAF-7 all of which exhibit narrow pore apertures (*ca*. 3.4 Å).

To enhance substrate diffusion and improve biocomposites conversion rates, Fa-Kuen Shieh’s group explored the expansion of the pore aperture and developed a synthetic approach to successfully encapsulate α-chymotrypsin (CHT) within MOF-74­(Zn).^19^ In this pore-expanded composite, the retention of biological function was demonstrated, whereas the activity of the narrow pore-sized CHT@ZIF-90 was undetectable. More recently, Huang et al.^18^ further demonstrated this concept, expanding the ZIF pore aperture in MAF-6 based biocomposites. Notwithstanding the validation of MOF pore expansion as a powerful strategy to improve biocatalysis, the more challenging enzymatic breakdown of polymers remains to be explored. In fact, current methods to access heterogeneous enzymatic depolymerization rely on the external adsorption of enzymes onto MOF surfaces or their *in situ* encapsulation into defect-rich MOF structures, both resulting in biocomposites where enzyme stability and reusability are compromised.^20–22^ Therefore, it is reasonable to develop MOF composites with large, accessible porosity capable of endorsing efficient depolymerization biocatalysis by easing the diffusion of large substrates.

Accessing new MOFs for biocomposite formation requires developing synthetic strategies that are compatible with biomolecule stability.^23^ In this context, we reported the Lewis acid-mediated encapsulation^24^ of proteins within MIL-100­(Fe),^25^ an original approach that overcomes the electrostatic limitations associated with ZIF-based mineralization methods. To exploit this chemical concept and generate composites with more open porosity for enzymatic depolymerization, we propose the direct synthesis of thealuminum-trimesate MIL-110­(Al),^26^ exhibiting open microporous channels (*ca*. 16 Å in diameter vs 5.5 and 8.8 Å pore apertures in MIL-100­(Fe)). Exploiting the *in situ* formation of aluminum-based MOFs offers several advantages: (1) aluminum is a nontoxic, inexpensive, and very abundant metal source; (2) Al^3+^ shows a rich aqueous chemistry with strong coordination ability toward organic linkers due to its high Lewis acidity, forming diverse secondary building units (SBUs) that assemble into various complex topologies and structures;^27,28^ and (3) Al-based MOFs demonstrate high hydrothermal stability, with decomposition temperatures around 450 °C due to the strong Al–O bonding conditions (e.g., MIL-53: 514 kJ mol^–1^),^28^ making them suitable candidates for industrial applications.^29^ Additionally, Al-compounds have been used as adjuvants in vaccine elaborations,^30^ and Al-MOFs have been recently proposed as possible replacement candidates.^31,32^ Notable examples of Al-MOFs include the archetypal MIL-96,^33^ MIL-100,^34,35^ and MIL-110,^26^ all synthesized by reacting aluminum nitrate with 1,3,5-benzenetricarboxylic (H_3_btc) or trimesic acid. Among these, MIL-110­(Al) stands out as the most promising candidate as it exhibits the largest porosity for optimal access of the substrates to the encapsulated protein. The structure [Al_8_(OH)_15_(btc)_9_] consists of a honeycomb lattice of octanuclear aluminum clusters that are linked through the trimesate ligand. MIL-110­(Al) features large hexagonal channels that can be exploited to direct biocatalysis, particularly in the enzymatic hydrolysis of polymeric substrates (Figure 1).

**Figure 1:**
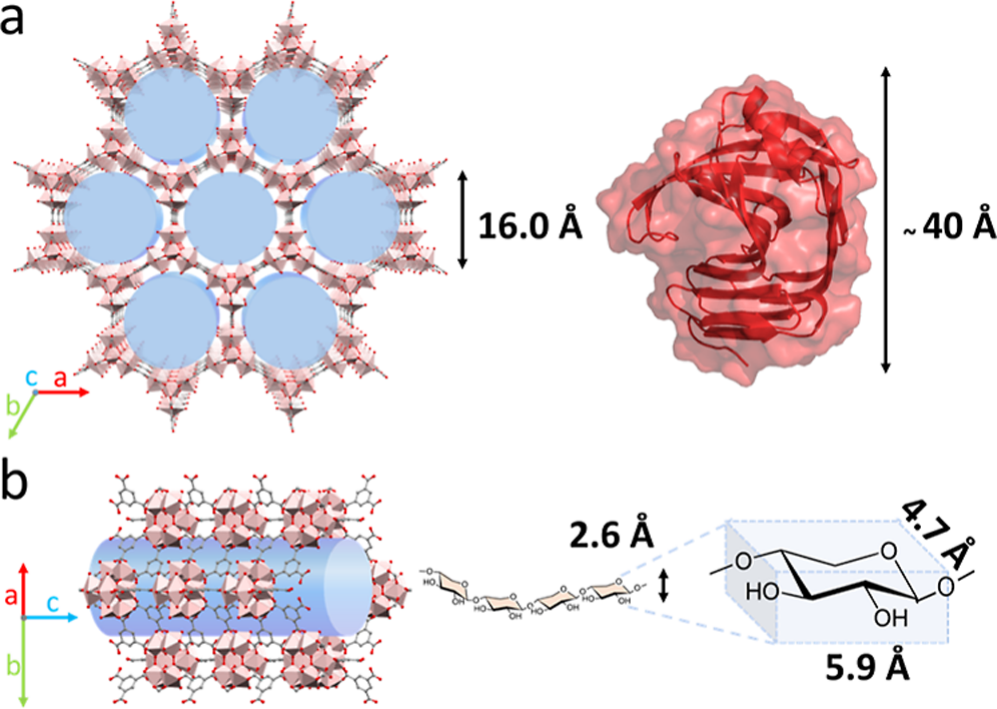
(a) Schematic representation of the aluminum­(III) trimesate MIL-110­(Al) structure of the (left) exhibiting channel-like porosity (blue) and the structure of a model xylanase protein (right) for size comparison. (b) Structure view of MIL-110­(Al) along the *c* axis displaying the pore and representation of the channel-directed accommodation of xylose polymer substrate. Sizes of the pore diameter and xylose polymer are indicated.

In this study, we developed a water-based, room temperature synthesis method for pure-phase MIL-110­(Al) nanoparticles. The method is compatible with the *in situ* encapsulation of proteins and has been validated for the continuous production of biocomposites. The suitable channeled architecture of MIL-110­(Al) is then tested for biocatalytic depolymerization, using as model protein a xylanase, Xyn3-CBM9—an endoacting enzyme that depolymerizes the linear polysaccharide xylan. Bioactivity of the confined Xyn3-CBM9 within MIL-110­(Al) is evaluated, demonstrating the successful application of a pore-directed strategy to regulate biocatalytic depolymerization within MOFs.

## 2 Results and Discussion

### 2.1 Green and Mild Synthesis of MIL-110­(Al)

A water-based, room temperature synthetic protocol compatible with bioentities was developed for the MIL-110­(Al) material. Previously reported hydrothermal syntheses were used as a reference to identify key synthetic parameters. For example, in a water-based, hydrothermal synthesis, Haouas et al.^36^ observed that pH is a critical parameter to obtain phase pure MIL-110­(Al). While MIL-110­(Al) is the thermodynamically stable product at pH ≈ 0, the formation of the MIL-96 phase is favored at pH ≈ 4 and above. Further studies demonstrated that both compounds could be isolated via an OH^–^ assisted method, with higher OH^–^ concentration favoring MIL-110­(Al) nanocrystals.^37^ However, these syntheses were all conducted in autoclaves, at elevated temperatures (*ca*. 150 °C and above) and for several days, making them unsuited for biomolecules. More recently, a green synthesis method was developed for MIL-110­(Al), however leading to a mixture containing the MIL-96­(Al) phase.^38^ To achieve a biocompatible synthesis of pure MIL-110­(Al), fine-tuning the pH in the range of 3.5 < pH < 8 is essential. Ideally, adjusting the concentration of NaOH and reactants within this range would promote the crystallization of a pure MIL-110­(Al) phase. Given the amphoteric nature of Al^3+^ ions in aqueous solution, it is expected that the monomer Al^3+^ prevails for pH < 3.5, whereas Al­(OH)_3_ forms and precipitates at higher pH until *ca*. 10.7, where Al­(OH)_4_^–^ becomes the dominant species.^39^ Only between pH ∼3.5 and ∼4.7, the formation of polyoxocations (POCs) containing diverse numbers of metal atoms is reported.^40–42^ It is expected that this narrow pH range would favor the formation of the pure MIL-110­(Al) phase (Figure 2a). To verify this hypothesis, we systematically varied the amount of added NaOH (from 3 to 8 equiv) while keeping the concentration of btc^3–^ constant, thus exploring a pH range from 3 to 7. In a general procedure, MIL-110­(Al) was synthesized by directly adding a 20 mM aqueous solution of btc^3–^ (10 mL) to a 40 mM aqueous solution of Al­(NO_3_)_3_·9H_2_O (10 mL) with constant stirring. A white precipitate formed immediately in all cases, and the mixture was allowed to stir for 1 h. All samples revealed the formation of Al-trimesate materials by FT-IR analysis (Figure S1), but only the sample obtained in the postulated optimal pH range (6 eq. NaOH) yielded the crystalline MIL-110­(Al) phase, as deduced by powder X-ray diffraction (PXRD) and sorption studies (Figure 2b,c, respectively). The rest resulted in amorphous Al-trimesate coordination network materials. TGA analysis of this crystalline phase exhibits a temperature profile with a sharp decomposition occurring at 500 °C, whereas the remaining inorganic residue confirms the purity of the material (experimental 39.0%, theoretical 35.6%) (Figure S2). N_2_ gas sorption isotherm profiles (Figure 2c) and transmission electron microscopy (TEM) images (Figure S3) further confirmed the growth of amorphous materials, forming undefined particles of *ca.* 25 nm and exhibiting negligible to minor sorption capacities with increasing NaOH equivalents (BET = 33, 103, 114, and 120 m^2^·g^–1^, respectively, for 3, 4, 5, 7, and 8 NaOH eq). On the other hand, MIL-110­(Al) obtained with 6 NaOH equivalents forms well-defined needle-like particles of *ca.* 350 nm length and reaches the expected N_2_ gas sorption capacity (BET = 1544 m^2^·g^–1^), in good agreement with reported values of hydrothermally synthesized MIL-110­(Al). This discrimination in the formation of MIL-110­(Al) is most probably due to (1) the pH-dependent stability window of Al^3+^ species in aqueous media (stable below pH ≈ 5)^43^ and (2) the thereof potentially formed Al_8_ octamer in solution. In fact, Perkins et al.^44^ reported the formation of Al_8_ octamers in sulfuric acid solution of Al­(OH)_3_. On the other hand, Lin et al.^29^ assumed that the presence of OH^–^ ions promotes the formation of μ_2_–OH connections (due to deprotonated btc^3–^ ligands) along the {101} direction. These factors explain the metastable nature of MIL-110­(Al)^45^ and support the required precise adjustment of the synthetic conditions to favor the formation of a pure phase material.

**Figure 2:**
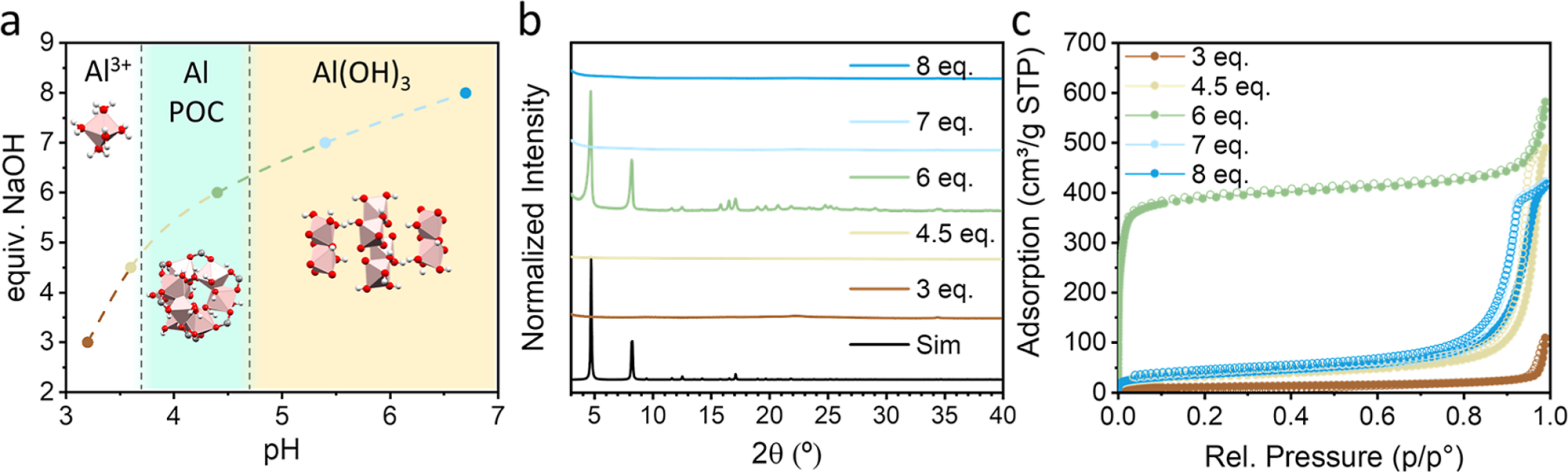
(a) Representation of added NaOH equivalents and resulting pH with the predominant aluminum species present in solution. (b) Experimental PXRD patterns of materials obtained with varying NaOH equivalents, compared to reference MIL-110­(Al) simulated pattern. (c) N_2_ gas sorption isotherms at 77 K of materials obtained with varying NaOH equivalents.

### 2.2 Exploiting MIL-110­(Al) Synthesis for Composite Formation

Once the synthetic protocol for phase pure MIL-110­(Al) was established, direct bioentity encapsulation was assessed using a variety of proteins including bovine serum albumin (**BSA**), glucose oxidase (**GOx**), hemoglobin (**Hb**), and lysozyme (**Ly**). These model proteins were selected as they display different isoelectric points (pI), allowing for the examination of the encapsulation efficiency of MIL-110­(Al) in diverse electrostatic scenarios. The inspection of these interactions at the MOF–protein interface is particularly relevant, as successful encapsulation fostered by spontaneous in situ growth of intensely studied MOFs, such as ZIF-8, strongly depends on the use of negatively charged biomolecules (low pI), an issue that hitherto has been circumvented by means of surface modification.^46,47^ In the present study, the use of the strong Lewis acid Al^3+^ is expected to promote effective interaction with all proteins, including those exhibiting low pI, directing the MOF growth onto the bioentity through a Lewis acid-mediated mechanism, as previously demonstrated by some of us with Fe^3+^ and the spontaneous growing of MIL-100­(Fe) biocomposites.^24^ BSA, GOx, Hb, and Ly composites were prepared following the general synthesis developed for MIL-110­(Al), adding the corresponding proteins to the Al^3+^ solution (see Supporting Information for details). Encapsulation efficiency and protein loading were determined by combining protein quantification of the supernatant and TGA analysis of the composites (Figure 3 and Supporting Information for details). All proteins were successfully encapsulated with high loading efficiencies (>97%) regardless of their pI, except for Hb (82%), as will be later discussed. In all cases, a negligible presence of weakly interacting enzymes on the MOF’s surface was detected upon ionic disrupting treatment (see experimental details), establishing that enzymes were entrapped within the MOF material (Table 1). Experimental TGA profiles depicted in Figure 3e revealed an additional weight loss in the 300–450 °C range in all protein@MIL-110­(Al) profiles, as compared to the empty MOF control profile. Protein loading was estimated considering the resulting inorganic residues (eq S1 in Supporting Information). Efficiencies and loadings were double-checked spectrometrically by quantification of the released protein after complete degradation of the composites in PBS using the BCA assay, revealing full recovery of the proteins.

**Figure 3:**
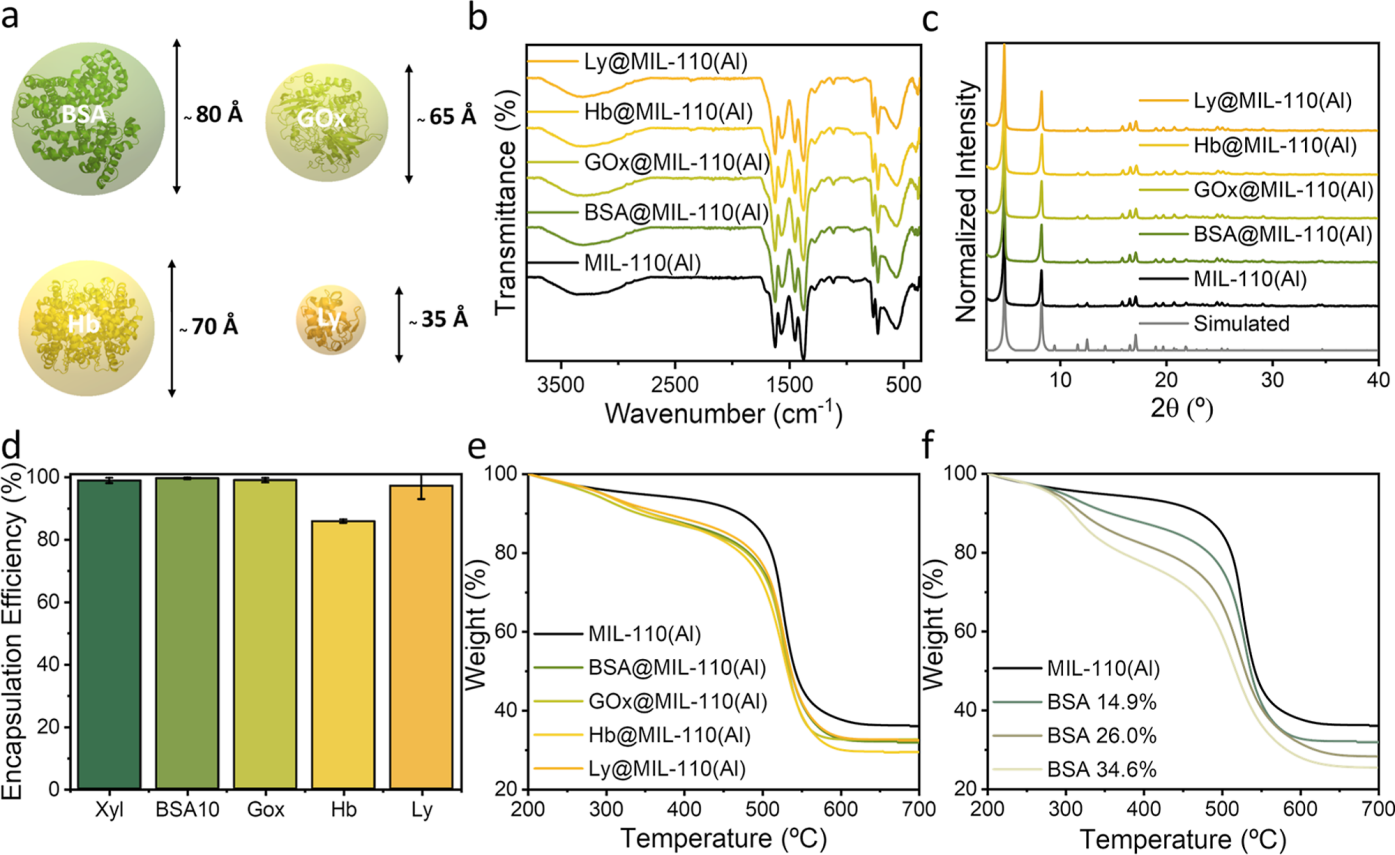
(a) Scheme of encapsulated proteins BSA, GOx, Hb, and Ly with their respective sizes. (b) FT-IR spectra, (c) experimental PXRD patterns, and (d) encapsulation efficiency of the different protein@MIL-110­(Al) composites with BSA, GOx, Hb, and Ly compared with empty MIL-110­(Al). (e) HR–TGA profiles of MIL-110­(Al) and composites and (f) HR–TGA profiles for BSA@MIL-110­(Al) with three different loadings. Measurements were conducted in a N_2_ atmosphere at a heating rate of 5 °C·min^–1^. All curves were normalized at 200 °C.

**Table 1:** Summary of MIL-110­(Al) Composites Obtained, with Electrostatic Characteristics of Encapsulated Proteins (pI), Encapsulation Efficiency, Loading Composition and Particle Size

material	protein pI	encapsulation efficiency [%]^a^	loading theor. [%]	loading exp. [%]^b^	size [nm]^c^
BSA1@MIL-110(Al)	4.5–4.8	99.6 ± 0.3	14.9	14.6	350
BSA2@MIL-110(Al)	4.5–4.8	99.5 ± 0.4	26.0	23.3	400
BSA3@MIL-110(Al)	4.5–4.8	99.2 ± 0.4	34.6	31.1	400
GOx@MIL-110(Al)	4.2 ^45^	99.0 ± 0.7	14.9	10.3	350
Hb@MIL-110(Al)	7.0 ^46^	85.9 ± 0.6	14.9	18.1	250
Ly@MIL-110(Al)	11.3 ^47^	97.2 ± 4.3	14.9	10.3	350
Xyl@MIL-110(Al)	≈5	98.9 ± 0.9	12.0	11.8	350

a As determined by Standard Bradford Assay.

b As estimated by TGA (% wprotein/wdry MOF).

c Particle size diameter measured by TEM (Figures S5 and S6).

Successful formation of MIL-110­(Al) was evidenced for all composites by FT-IR analysis, where the characteristic bands of the MIL-110­(Al) material—the very intense ν­(COO^–^) vibrations^48^ overlap with the most representative bands of the proteins (amide I, II, and III) (Figures 3b and S4). PXRD analysis confirmed successful MIL-110­(Al) formation for all proteins, retaining the phase purity of MIL-110­(Al), with no evidence of competing MIL-96 or MIL-100 phase formation (Figure 3c). TEM analysis reveals the formation of needle-like crystals of ca. 350 nm in size, whereas in the case of Hb@MIL-110­(Al), smaller nanoparticles of 250 nm are formed (Figure S5). Control over the protein loading was proven by preparing BSA@MIL-110­(Al) composites with three different loadings (14.9, 26.0, and 34.6% based on % wProtein/wdry MOF, Figure 3f). No significant differences in encapsulation efficiency, composite purity, and crystallinity were observed, whereas an increase in particle size to 400 nm was noticed upon increasing the BSA loading (Table 1, Figure S6). Related with the different encapsulation efficiency detected in the **Hb@MIL-110­(Al)** composite, one can consider the high pI of this protein as a detrimental factor that precludes MOF growth to some extent, as previously observed with negatively charged biomolecules for successful, spontaneous ZIF-8 encapsulations.^46,47^ However, this effect is not observed in the encapsulation of **Ly**, containing more basic residues than **Hb**. Our hypothesis is that Al-Hb conjugates form and precipitate. This is supported by TGA and protein loading analysis since a lower percentage of the remaining inorganic residue at temperatures above MIL-110­(Al) decomposition (>550 °C) points to the event of missing Al-clusters defects in the Hb-composite, which are only observed in this composite.

### 2.3 Scaling up the Synthesis of MIL-110­(Al) and Composites

In view of the general applicability of our MIL-110­(Al)-encapsulation methodology, including potential interest in an industrial scheme, we investigated the possibility of scaling up production. To achieve this, our Lewis acid-mediated protocol was adapted to a continuous flow setup (Figure 4a). Two precursor solutions were prepared separately, S1 containing a 40 mM Al­(NO_3_)_3_ solution with or without BSA (10 mg·mL^–1^) and S2 consisting of a 20 mM H_3_btc solution with 6 eq. of NaOH. The flow of these precursor solutions was carried out using a multichannel peristaltic pump with a continuous flow of 2 mL·min^–1^, mixed with a Y-connector and then passed through a coiled reactor. This coiled reactor enhances the mixing by producing different fluid velocities with the curvature and creating counterrotating vortices alongside the liquid flow.^49^ This simple and inexpensive setup has been proven to enhance reproducibility in MOF synthesis, particularly in ZIF-8 production.^50,51^ Conditions for MIL-110­(Al) pure phase formation under continuous flow were optimized by tuning the Al/H_3_btc ratio from the 2:1 employed in the batch synthesis, to the adjusted 4:1 ratio (Figure 4b). A similar trend was observed when solutions S1 and S2 were added simultaneously and mixed in a vial (Figure S7), confirming the requirement of excess Al^3+^ for the formation of a phase pure material. Structural and compositional analyses of the empty and BSA-loaded materials obtained in continuous-mode were carried out by PXRD, TGA and TEM analyses (Figure 4c–e) revealing the high quality and preservation of properties of the materials obtained in a continuous production scheme.

**Figure 4:**
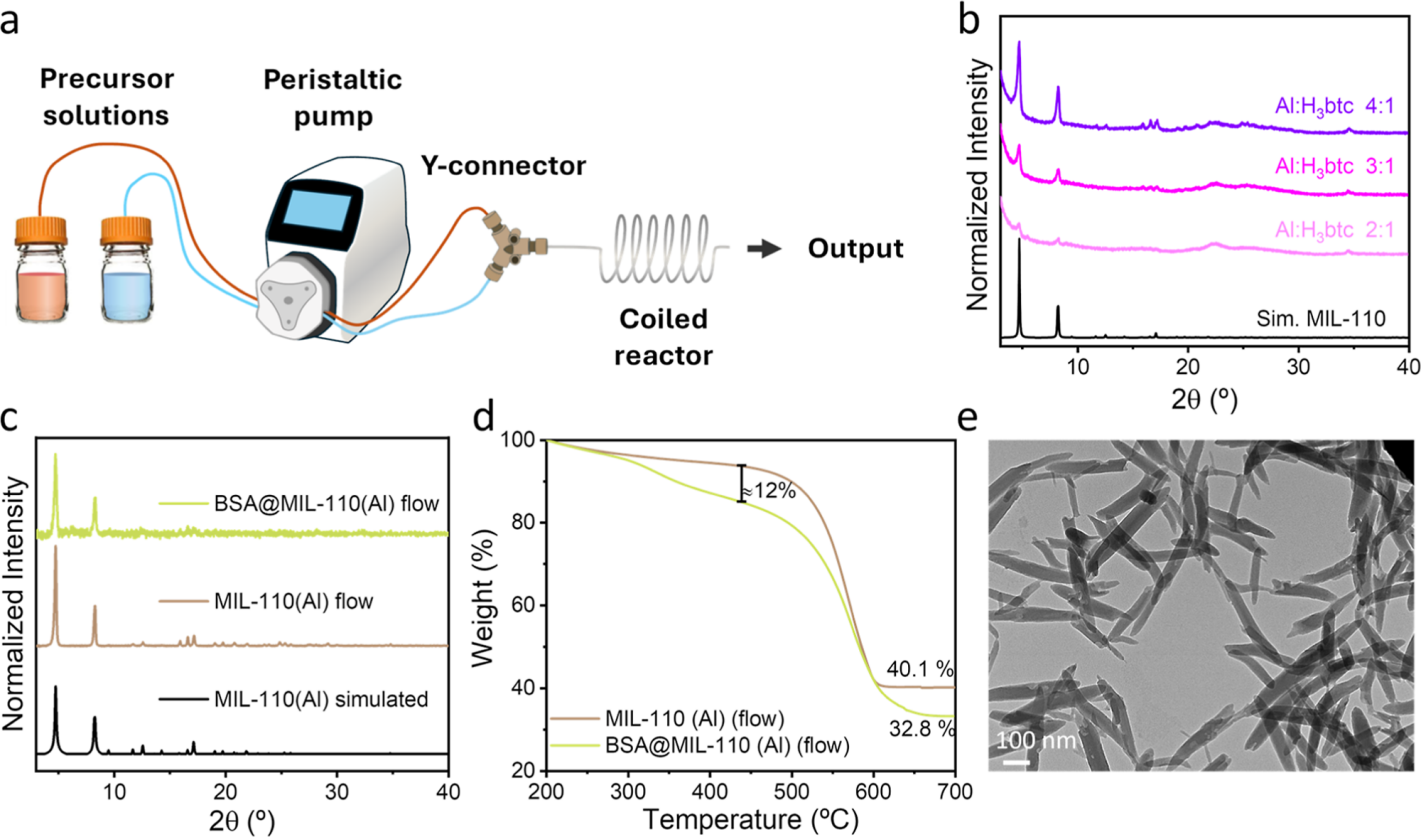
(a) Scheme of the setup for the continuous flow synthesis of MIL-110­(Al). (b) PXRD patterns of the materials obtained with different Al/H_3_btc ratios in a continuous flow setup. (c) PXRD patterns of the obtained BSA@MIL-110­(Al) composite compared to the MIL-110­(Al) of the same batch. (d) TGA of control MIL-110­(Al) and BSA@MIL-110­(Al) synthesized in a continuous flow setup. (e) TEM image of the obtained BSA@MIL-110­(Al) composite.

### 2.4 Xylanase Entrapment within MIL-110­(Al)

Once the method to obtain MIL-110­(Al) composites was set up, including different production scales, a target model enzyme active in depolymerization reactions, xylanase Xyn3-CBM9 expressed by Talens-Perales et al.,^52,53^ was encapsulated (Figure 5a). This specific xylanase was selected to provide a proof of concept of effective depolymerization biocatalysis using MOF composites. The target **Xyl@MIL-110­(Al)** composite was formed obtaining high efficiencies (*ca.* 99%) with the expected loading (*ca*. 12%) (see Table 1). TEM analysis of the Xyl@MIL-110­(Al) composite disclosed the formation of monodisperse, needle-like nanocrystals of *ca.* 300–400 nm length and *ca.* 50–100 nm width (Figure 5b). This morphology corresponds with the observed in the control empty nanoparticles (Figure S5) and fit with the hexagonal needle-like single crystals (*ca.* 3 × 3 × 10 μm) of MIL-110­(Al) obtained via the solvothermal synthesis route.^26^ It is concluded that the confinement of Xyl within the MIL-110­(Al) does not impact the nanoparticle morphology, as expected for mineralized samples, where the scaffold grows around the protein. FT-IR, PXRD, and TGA characterizations confirmed successful composite formation, retaining the phase purity of MIL-110­(Al) (Figure 5c–e).

**Figure 5:**
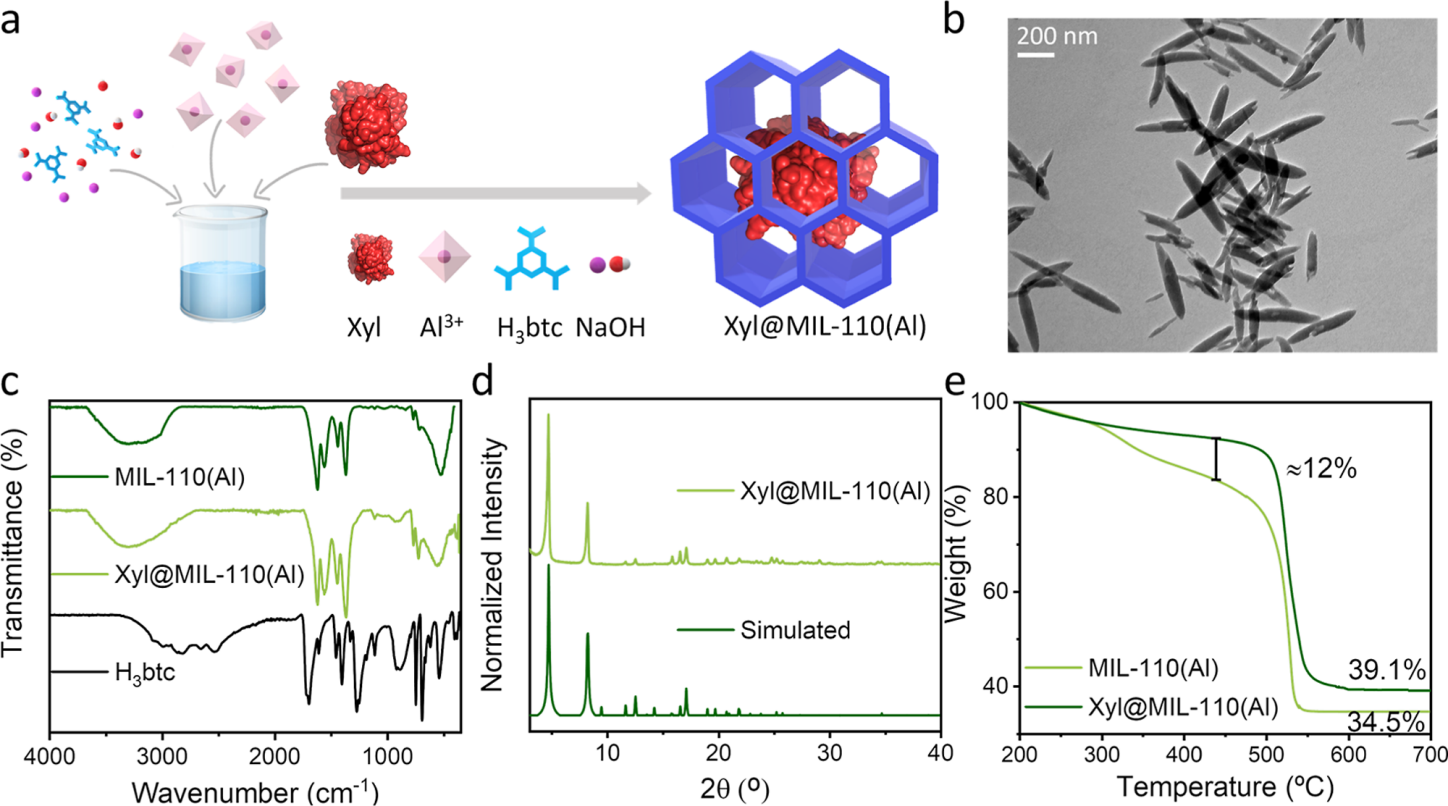
(a) Schematic representation of the one-pot, Lewis-acid mediated synthesis and the resulting Xyl@MIL-110­(Al) composite. Characterization of the Xyl@MIL-110­(Al) composite by (b) TEM, (c) FT-IR, (d) PXRD, and (e) TGA. MIL-110­(Al) and H_3_btc ligand are represented as control empty MOF and precursor, respectively.

Xyn3-CBM9 encapsulation was also evaluated using qualitative protein analysis by means of Sodium Dodecyl Sulfate–Polyacrylamide Gel Electrophoresis (SDS-PAGE). Three samples were analyzed, including the initial free protein solution used for encapsulation (lane A), the protein fraction released after ethylenediaminetetraacetic acid (EDTA) treatment of encapsulated Xyl@MIL-110­(Al) (lane B), and an SDS wash fraction following incubation of xylanase with preformed MIL-110­(Al) (lane C), to assess potential surface adsorption (Figure 6). A prominent band at ∼44 kDa, corresponding to the theoretical size of the Xyn3-CBM9 xylanase, was observed for the tested samples of free Xyl and the released from Xyl@MIL-110­(Al) (respectively, lanes A and B), corroborating the efficient encapsulation during in situ MOF formation. In contrast, no detectable signal was observed in the Xyl incubated with preformed MIL-110­(Al) (lane C). This experiment confirms the inner location of the enzyme within MIL-110­(Al) upon in situ synthesis and reveals the absence of detectable enzyme superficially adsorbed on the MOF external surface.

**Figure 6:**
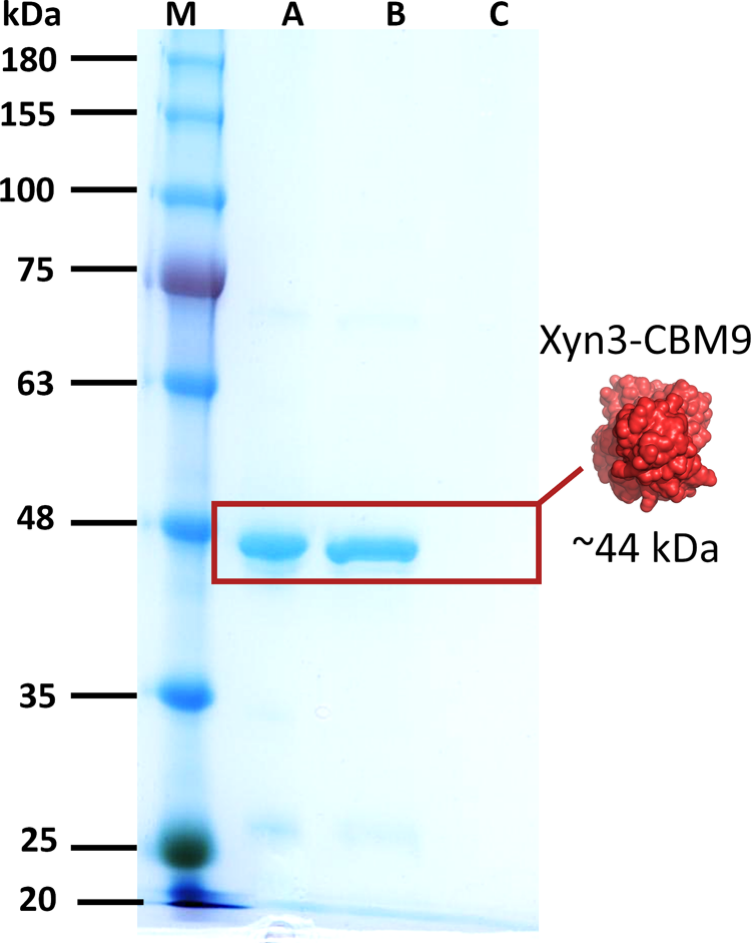
Electrophoretic analysis to confirm enzyme encapsulation in SDS-PAGE. **Lane M**, protein standards; **lane A**, initial Xyl solution used for encapsulation–monomeric molecular weight ∼44 kDa; **lane B**, protein fraction recovered following EDTA-induced release from Xyl@MIL-110­(Al) encapsulates; **lane C**, protein content in the wash fraction after xylanase incubation with preformed MIL-110­(Al) (surface adsorption control).

### 2.5 Channel-Directed Catalytic Activity of Xylanase@MIL-110­(Al): Xylan Depolymerization

The hybrid Xyn3-CBM9 obtained by molecular engineering combines the xylanase from Thermobacillus xylaliniticus with the carbohydrate binding domain of CBM9 from Thermotoga maritima, which eases the enzyme binding to organic residues in xylan and cellulose for efficient depolymerization. In compatible composite conditions (buffered solution at pH 5 and 65 °C), free Xyn3-CBM9 shows high activity toward xylose hydrolysis, which makes this xylanase an optimal model to test the effect of enzyme confinement within MOFs for challenging depolymerization reactions.

Our aim was to investigate the extent of catalytic xylanase performance after encapsulation within MIL-110­(Al) following our Lewis acid-mediated strategy. For this, Xyl@MIL-110­(Al) and the free enzyme were incubated for 10 min in acetate buffer (50 mM, pH = 5, 60 °C) with a 1% water-soluble beechwood xylan. The reaction was terminated by a rapid cooling. Samples were then centrifuged, and the supernatant was analyzed (see Experimental Section for details). A delay in enzymatic hydrolysis was observed for Xyl@MIL-110­(Al) (*ca*. 7.3 vs 13.5 μmol·min^–1^·nmol^–1^ for the free enzyme), resulting in an effectiveness factor of 0.54, as expected for a more restricted substrate accessibility (i.e., substrates need to diffuse inside the scaffold through the pore channels to interact with the enzyme, Figure 7a). It is important to emphasize that activity of the free enzyme was fully recovered after composite degradation and release in PBS, revealing that the encapsulation process does not directly affect the enzyme’s bioactivity (Figure S8). Noticeably, the observed composite activity value may be improved by tuning the catalytic conditions, for instance, by increasing the reaction time, until the best operational conditions are found. Instead, we have focused our efforts on revealing more important benefits of the MOF-composite regarding catalytic depolymerization since the observed slowdown of the catalytic process does not affect the enzyme productivity on a large timescale.

**Figure 7:**
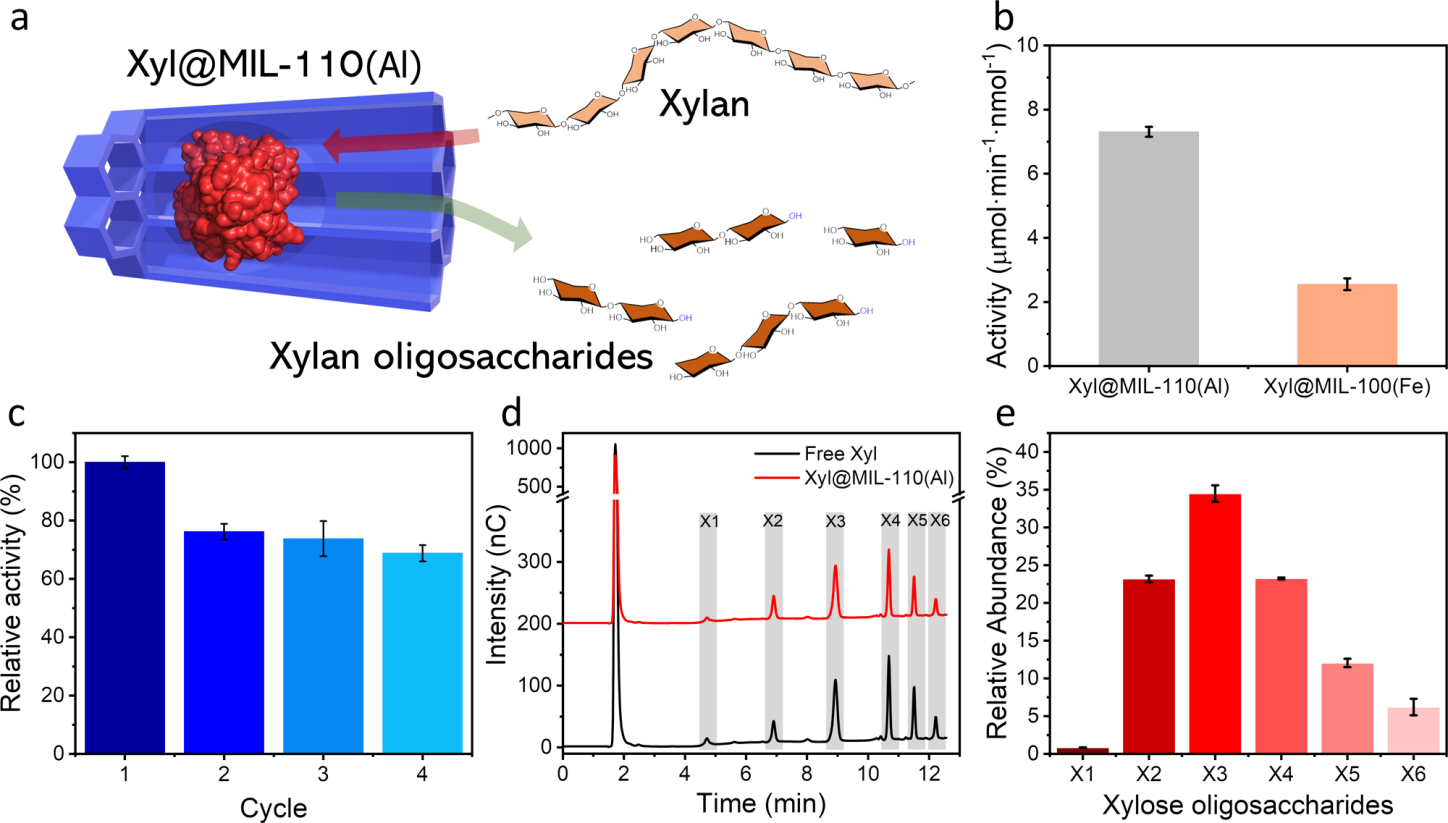
(a) Schematic representation of channel-directed mass transport inside Xyl@MIL-110­(Al) during catalytic depolymerization of xylan into xylooligosaccharides. (b) Enzymatic activities of encapsulated Xyl in MIL-110­(Al) and MIL-100­(Fe). (c) Activity retention after consecutive depolymerization. (d) Chromatographic profiles of the depolymerization products from Xyl and Xyl@MIL-110­(Al). Peaks indicated as X1–X6 correspond to the number of xylose units of the products. (e) Relative abundance of the products catalyzed by Xyl@MIL-110­(Al) according to their xylose units.

To evidence the exceptional channel-directed substrate accessibility offered in the MIL-110­(Al) scaffold, we investigated the same enzymatic hydrolysis reaction in the more sterically restricted scenario offered by the MIL-100­(Fe) scaffold (Figures S9 and S10).^35,54^ This hierarchical, mesoporous Fe-trimesate material exhibits two distinct large cages (diameters of ca. 25 and 29 Å) that, in the absence of channels, are interconnected through pentagonal and hexagonal windows (respective diameters of 5.5 and 8.8 Å). Catalytic experiments conducted for **Xyl@MIL-100­(Fe)** under the same conditions revealed a significant 5-fold decrease in activity (*ca*. 2.6 μmol·min^–1^·nmol^–1^) with an effectiveness factor of only 0.19, in agreement with a more restricted diffusion of xylan polymers through the scaffold (Figure 7b). This significant difference in activity evidences the effective channel-directed performance enabled in the open MIL-110­(Al) structure.

One of the main advantages of Xyn3-CBM9 immobilization is the ability to overcome the single use limitation of the free enzyme, which hampers its cost-effective exploitation. Heterogenous catalytic performance of **Xyl@MIL-110­(Al)** was tested over consecutive depolymerization reactions retaining >75% of the initial Xyn-CBM9 activity after four cycles (5.6 μmol·min^–1^·nmol^–1^) (see Experimental Section for details). The observed decrease in activity may be attributed to a partial enzyme deactivation resulting from the confined state or to some blocking of the accessible channels after consecutive xylan addition. One should also relate such continuous drop of activity with a plausible loss of Xyl@MIL-110­(Al) composite catalyst after consecutive centrifugation and washing processes, provided that a similar constant decay of activity was observed in the less favored Xyl@MOF-100­(Fe) composite (Figure S12). Overall, the use of Xyl@MOF-110­(Al) results in an effective 3-fold increase of activity with respect to the free, nonrecoverable Xyn-CBM9 enzyme, as consequence of the depolymerization products obtained over consecutive cycles.

### 2.6 Analysis of Xylooligosaccharides Production

Hydrolytic xylan depolymerization yields a variety of xylooligosaccharides (XOS), consisting of a certain number of d-xylose units (between 1 and 6) linked by β-1,4-glycosidic bonds. These hydrolyzed XOS products have found their use as prebiotics, in pharmaceuticals—antiviral and anticancer medications—and above all, in the food industry,^55^ this last with an interest over XOS polymerization degree of 2–4 (xylobiose, xylotriose, and xylotetrose).^56^ In search of an efficient and cost-effective xylanase-based bioprocess, we investigated the production of XOS from the encapsulated xylanase in Xyl@MIL-110­(Al) using anionic exchange chromatography (see the Experimental Section for details). After 10 min of incubation, oligomers ranging from 2 to 6 residues in length were detected. No discernible differences in the composition of XOS produced by free or encapsulated Xyn3-CBM9 within MIL-110­(Al) were identified (Figure 7d). Analysis of the relative abundance in Figure 7e shows that encapsulated Xyn3-CBM9 in MIL-110­(Al) mainly produces trisaccharides (X3, *ca.* 35%) followed by an equal amount of di- and tetrasaccharides (X2 and X4, *ca.* 23%), whereas free Xyn3-CBM9 tends to form less X2 (*ca.* 16%). This analysis confirms that the catalytic ability of Xyn3-CBM9 is not affected by the encapsulation within MIL-110­(Al). It is observed that Xyn3-CBM9 confinement within MIL-110­(Al) facilitates a higher degree of xylose hydrolysis, which may be related with the channel-like scaffold favoring a local accumulation of substrates in the enzyme surroundings, produces a higher degree of xylose hydrolysis, which may be related with the channel-like scaffold favoring a local accumulation of substrates in the enzyme surroundings.

## 3 Conclusions

This work proposes the green, mild synthesis of MIL-110­(Al) nanoparticles, highlighting the importance of pH control for successful pure phase formation under these conditions. Our protocol is compatible with the Lewis acid-mediated encapsulation of different proteins reaching high efficiencies and with control over protein loading, ensuring the spontaneous growth of composites regardless of the protein nature, therefore surpassing the electrostatic linmitations encountered in zeolitic *in situ* encapsulation. The synthetic protocol has been validated for the continuous production of pure-phase protein@MIL-110­(Al) composites, meeting the requirements for industrial production. The structural features of the MIL-110­(Al) composites, with exceeding pore apertures and channels, were evaluated in a challenging enzymatic depolymerization reaction. A model, tailored xylanase, Xyn3-CBM9, with activity toward xylan polymer hydrolysis, was confined within MIL-110­(Al), and the production of reducing sugars from xylan depolymerization was monitored. It is observed that employing MIL-110­(Al) for xylanase immobilization facilitates substrate diffusion compared to the sterically restricted mesoporous MIL-100­(Fe) scaffold. Overall, the channel-directed xylan depolymerization occurring within MIL-110­(Al) over successive catalytic cycles results in a 3-fold enhanced catalytic performance of Xyn3-CBM and a higher degree of xylose hydrolysis. Although the reaction mechanism of embedded enzymes is not yet understood in detail, our results provide insights into how to control biocatalysis in solid matrices, offering effective and affordable opportunities for challenging polymer biodegradation processes.

## 4 Experimental Section

### 4.1 Characterization Techniques

FT-IR spectroscopy was performed on an ALPHA II spectrometer (Bruker) in the range 400–4000 cm^–1^ using an ATR accessory with a diamond window. XRPD patterns were obtained using an X-ray diffractometer (PANalytical Empyrean) with copper as a radiation source (Cu–Kα 1.5418 Å) operating at 40 mA and 45 kV and equipped with an X’Celerator detector. Measurements were collected on quartz capillaries or on a high-throughput screening platform (HTS). Protein quantification was determined following the Bradford Assay method or bicinchoninic acid (BCA) assay kit provided by Thermo Scientific Pierce. UV–vis spectra (with single wavelength analysis at λ = 562 nm) were recorded with a Microplate spectrophotometer Multiskan Sky (Thermo Scientific). TEM images were captured using a high contrast transmission electron microscope of either 100 Kv with a digital AMT camera with 8 Mpx or 120 Kv with a digital camera CMOS EMSIS XAROSA with 120 Mpx. TGA profiles were collected using TGA 550 (TA Instruments) at temperatures from 25 to 700 °C under N_2_. The temperature was stabilized at 50 °C before the measurement. The heating rate was established in the high-resolution mode (HR), starting at 5 °C·min^–1^ and decelerating when significant weight variation was measured. N_2_ isotherms were measured with a TRISTAR-2 apparatus (Micromeritics) at 77 K. Samples were treated under vacuum at 100 °C for 1 h prior analysis.

### 4.2 Encapsulation Efficiency and Loading

Encapsulation efficiency was assessed by measuring the differences in the protein concentration of the supernatant before and after encapsulation using a Standard Bradford Assay. Weakly interacting proteins attached at the surface were first discharged by analyzing the supernatant of a composite treated with sodium dodecyl sulfate (SDS) solution (5%) for 30 min at 60 °C. Loading was estimated from TGA analysis by direct comparison of the inorganic residues at 500 °C using normalized thermal profiles of protein@MIL-110­(Al) and control MIL-110­(Al). All experiments were performed in triplicates.

### 4.3 Protein Release

Protein release has been achieved by exposing the composite to PBS (100 mM, pH = 7) at room temperature in a thermoshaker (300 rpm) during *ca*. 24 h or with EDTA (500 mM, pH = 8) for 10 min.

### 4.4 Electrophoretic Analysis of MOF Encapsulation in SDS-PAGE

First, Xyl@MIL-110 (Al) was dissolved in EDTA (500 mM, pH = 8) for 10 min and centrifuged (13,300 rpm, 5′). Then 40 μL of the remaining liquid solution was drawn to mix with 40 μL of the formulated Laemmli sample buffer (sample buffer, Laemmli 2× concentrate, containing 4% SDS, 20% glycerol, 10% β-mercaptoethanol, 0.004% bromophenol blue, and 0.125 M Tris HCl, pH ∼ 6.8) and heated at 95 °C in water for 5 min. A total of 40 μL of the mixture was electrophoresed on SDS-PAGE (4.5% polyacrylamide stacking gel Tris HCl, pH ∼ 6.8, resolving gel 10% Tris HCl, pH ∼ 8.8) at 100 V. After electrophoresis, the gels were stained with BlueSafe (NZYtech) and image captured with Amersham ImageQuant 800 (Cytiva).

### 4.5 Evaluation of Xylanase Activity: Depolymerization of Xylan

Xylanase activity assays were conducted in triplicates by combining 360 μL of substrate solution (comprising 1% beechwood xylan dissolved in 50 mM acetate buffer at pH 5.0) with 40 μL of Xyn3-CBM9 (free or encapsulated enzyme forms were adjusted to ensure a linear response according to loadings) and subsequently incubated at 60 °C for 10 min. The end of the enzymatic reaction was dictated by rapid cooling of the reaction tubes on ice. Control reactions, without enzyme, were carried out for each assay condition. To quantify the released reducing sugars, 100 μL of 3,5-dinitrosalicylic acid (DNS) reagent was added to 200 μL of supernatant from reaction tubes (previously centrifuged during 10 min at 14,000 g at 4 °C) following a 10 min boiling step. Postincubation, 900 μL of Milli-Q water was added into the reaction mixture, followed by centrifugation. Optical density measurements were conducted at 540 nm in 96-well plates, with 300 μL of the supernatant transferred using a PowerWave HT spectrophotometer manufactured by BioTek Instruments (Winooski, VT, USA). The feasibility of MOF reuse was assessed through consecutive cycles of xylose hydrolysis. After each reaction batch, the Xyn3-CBM9@MIL-110­(Al) catalyst was recovered via centrifugation and subsequently reconstituted in 40 μL of buffer C and 360 μL of substrate solution to initiate a new reaction cycle. In a comparative study of activity, Xyl@MIL-100­(Fe) and Xyl@MIL-110­(Al) catalyst samples were dispersed in the exact volume of xylanase employed in the synthesis, to maintain the same pro concentration.

### 4.6 XOS Analysis

Analysis of XOS was carried out by ion exchange chromatography using a Dionex (Thermo Fisher Scientific) instrument equipped with a CarbonPac PA100 column and a pulsed amperometric detector (Dionex, Thermo Fisher Scientific). Xylose and XOS, from two to six units, were used as chromatographic standards. Prior to analysis, samples were standardized to equal levels of reducing sugars.

## Supplementary Material



## Data Availability

The characterization data files are available at Zenodo open repository at https://doi.org/10.5281/zenodo.15311199.

## Notes

Materials, synthetic methods, FT-IR spectra, detailed TGA analysis, TEM images of biocomposites, scale-up setup, characterization of MIL-100­(Fe), catalytic activities, and XOS formation (PDF)
