# Supplementary material for: Channel-Directed Enzymatic Depolymerization within a Metal–Organic Framework
Source: ACS Appl Mater Interfaces. 2025 May 1;17(20):29729–39. doi: 10.1021/acsami.5c04137 (PMC12128026; doi:10.1021/acsami.5c04137)
Supplement: Supplementary file 1 [file am5c04137_si_001.pdf]

Supporting Information

# Channel-directed enzymatic depolymerization within a metal-organic framework

*Jana Glatz[a] ‡, Jesús Cases Díaz[a] ‡, Jorge Salinas-Uber[a], David Talens-Perales[b], Julio Polaina[b], and Mónica Giménez-Marqués[a] **

[a] J. Cases Díaz, Dr. J. Glatz, Dr. J. Salinas-Uber, Dr. M. Giménez-Marqués
Universidad de Valencia - Instituto de Ciencia Molecular, Catedrático José Beltrán Martínez 2, 46980 Paterna, Spain

[b] Dr. D. Talens-Perales, Prof. J. Polaina
Instituto de Agroquímica y Tecnología de Alimentos (CSIC), Avda. Catedrático Agustín Escardino 7, 46980 Paterna, Spain

*monica.gimenez-marques@uv.es

**Materials**

All chemicals are commercially available and used as received. Al(III) chloride, benzene 1,3,5-tricarboxilic acid (BTC), tris(hydroxymethyl)aminomethane (Tris), calcium chloride, sodium hydroxide, Bovine serum albumin (BSA), Glucose oxidase (GOx) and Haemoglobin (Hb) were purchased from Sigma-Aldrich (Europe), Lysozyme (Ly) from Alfa Aesar (Europe). Di-sodium hydrogen phosphate and sodium dodecyl sulfate (SDS) were purchased from PanReac AppliChem (Germany), hydrochloric acid from AnalR NORMAPUR (France), iron(II) chloride tetrahydrate from VWR Chemicals (Europe) and trichloroacetic acid from Alfa Aesar (Europe). Milli-Q water was obtained from a Millipore Milli-Q system. Beechwood xylose was obtained from Sigma-Aldrich and the XOS standards from Megazyme.

**Synthesis of MOF and protein@MOF composites.**

***Synthesis of MIL-110(Al).*** In an open vial, 10 ml of 1,3,5-benzenetricarboxylic acid, $H_3btc$ (0.2 mmol) and sodium hydroxide (NaOH) (1.2 mmol) dissolved in double-distilled $H_2O$ ($ddH_2O$) were added to 10 ml of aluminium nitrate nonahydrate, $Al(NO_3)_3 \cdot 9H_2O$ (0.4 mmol) in $ddH_2O$.

A white suspension immediately formed and was left for 1 h at room temperature under vigorous stirring. A crystalline powder was collected by centrifugation (5 min at 8000 rpm) and then suspended and rinsed three times in dd$H_2O$. The material was air dried at room temperature. (Yield: 75-80% analysed via HPLC).

***Synthesis of BSA@MIL-110(Al) composites.*** **Protein@MIL-100(Al)** composites were obtained following the one-pot procedure established for MIL-110 but in presence of Bovine Serum Albumin (BSA), Glucose oxidase (GOx), Haemoglobin (Hb), Lysozyme (Ly) or xylanase (Xyl). For each composite, 10 mg of the corresponding protein was added to the $Al(NO_3)_3 \cdot 9H_2O$ aqueous solution. In the case of BSA encapsulation, in addition to form **BSA10@MIL-110(Al)** by adding 10 mg, two more composites were prepared **BSA20@MIL-110(Al)** and **BSA30@MIL-110(Al)**, by correspondingly adding 20 and 30 mg of BSA. In all cases a white suspension immediately formed and was left for 1 h at room temperature under vigorous stirring. Aliquots of the synthesis were stored as water suspension for further analyses. The materials were dried on air at room temperature.

***Continuous Flow biocompatible synthesis of MIL-110(Al) and composites.*** Two precursor solutions were individually prepared: S1 by mixing a 40, 60 or 80 mM solution of $Al(NO_3)_3$ and S2 consisting of 20 mM btc with 6 eq. of NaOH. When BSA was added (1 mg/mL), 80 mM $Al(NO_3)_3$ was employed. These precursor solutions were pumped using a multichannel peristaltic pump with a continuous flow of 2 $mL \cdot min^{-1}$ and a tube of 0.8 mm of inner diameter. Solutions were mixed with a Y-connector and then passed through a coiled reactor. After passing through the coil, the product was collected by centrifugation (6000 rpm 5min) and washed with ddH2O three times before being dried at room temperature (Yield:78% based in the amount of BTC; STY: 0.792 $Kg \cdot L^{-1}day^{-1}$).

***Simultaneous addition synthesis of MIL-110(Al).*** Two precursor solutions were individually prepared: S1 by mixing $Al(NO_3)_3$ solutions with 40, 60 or 80 mM (10 mL) and S2 consisting of 20 mM btc with 6 eq. of NaOH (10 mL). These precursor solutions were pumped using a multichannel peristaltic pump with a continuous flow of 2 mL·min–1 and a tube of 0.8 mm of inner diameter and dumped into a vial, were both mixed under stirring. After complete addition of both solutions, the product was collected by centrifugation (6000 rpm 5min) and washed with ddH2O three times before being dried at room temperature.

***Synthesis of Xyl@MIL-100(Fe).*** **Xyl@MIL-100(Fe)** was synthesized following the previously reported procedure.[1] Briefly, 20 mL of ligand solution containing btc and Tris base (0.4 mmol and 2.0 mmol, respectively) was prepared with dd$H_2O$ and adjusted to pH 7.5. After degassing, iron(II) chloride tetrahydrate, $FeCl_2 \cdot 4H_2O$ (0.4 mmol) was added. 10 mg of Xyl was added to

the ligand solution and put under stirring. Then, 20 mL of solution of iron(III) chloride hexahydrate, $FeCl_3 \cdot 6H_2O$ (0.4 mmol) in $ddH_2O$ was degassed and added to the ligand-Xyl solution at 20 mL/h. After completion of the reaction, the product was centrifuged at 8000 rpm for 10 min and washed three times with $ddH_2O$. After the synthesis, the supernatant was collected by centrifugation, and protein concentrations calculated from the supernatant. Aliquots of the synthesis were stored as water suspension for further analyses. The material was dried on air at room temperature.

**Cloning and production of Xyn3-CBM9.** Xyn3-CBM9 used as a reporter enzyme was obtained as a new gene construction based on Xyn3 described in Talens-Perales et al., (2020)[2]. The gene was constructed from the plasmid TmLac-CBM9 (Miguez Amil et al., 2020)[3] replacing the TmLac gene by the Xyn3 gene by digesting the gene and the receptor plasmid with SacI and SalI restriction enzymes (ThermoScientific). DNA fragments were ligated with T4 ligase (ThermoScientific) and the resulting plasmid (Xyn3-CBM9 PQE80L) was transformed in *E. coli* Rosseta2 (Stratagene). Xyn3-CBM9 was obtained from cell lysates were prepared from *E. coli* culture caring the Xyn3-CBM9 PQE80L plasmid, cultivated at 37°C until reaching an optical density (OD600) of 0.6, followed by induction with 1 mM IPTG at 37 °C for 5 hours. Cells were collected by centrifugation sonicated in buffer A (20 mM phosphate buffer at pH 7.4, 20 mM imidazole, and 500 mM NaCl). Subsequently, protein extract was obtained through centrifugation at 12,000×g for 25 minutes and subjected to nickel-affinity chromatography, employing a 1 mL HisTrapFF crude column (Cytiva) integrated into an AKTA-Purifier System (Cytiva). The elution was conducted using buffer B (20 mM phosphate buffer at pH 7.4, 500 mM imidazole, and 500 mM NaCl). Fractions revealing xylanase activity were dialyzed against buffer C (20 mM Tris–HCl at pH 7 and 50 mM NaCl). Protein concentration in the eluted fractions was determined in a NanoDrop spectrophotometer (Thermo Fisher).

**Characterization of MIL-110(Al) nanoparticles obtained under biocompatible conditions.**

**FT-IR Spectroscopy**

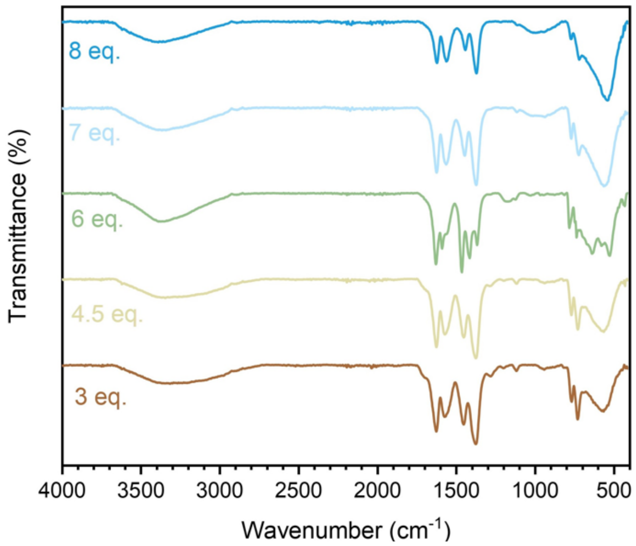


**Figure S1.** FT-IR spectra of Al-btc materials synthesized using different equivalents of NaOH.

**Thermogravimetric Analysis (TGA)**

MIL-110(Al) shows a small and very gradual decrease (*ca*. 5 %) in weight up to 500 ºC which can be attributed to the removal of the three structural water molecules. Then, an almost instant weight loss leading to *ca*. 39 % of residual mass can be observed associated with the btc linker release and structure collapse. The remaining inorganic residue (*ca.* 39.0 %) agrees with the theoretically expected value (35.5 %) and suggests that the material lacks significant defects.

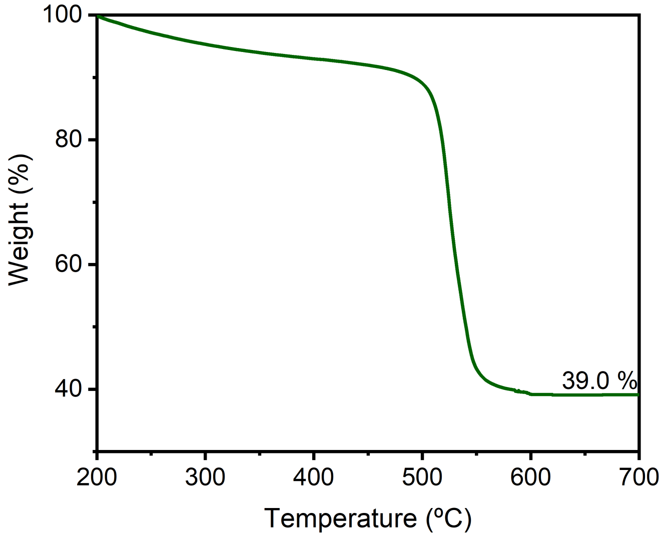


**Figure S2.** TGA analysis of MIL-110(Al) material obtained with 6 NaOH equivalents.

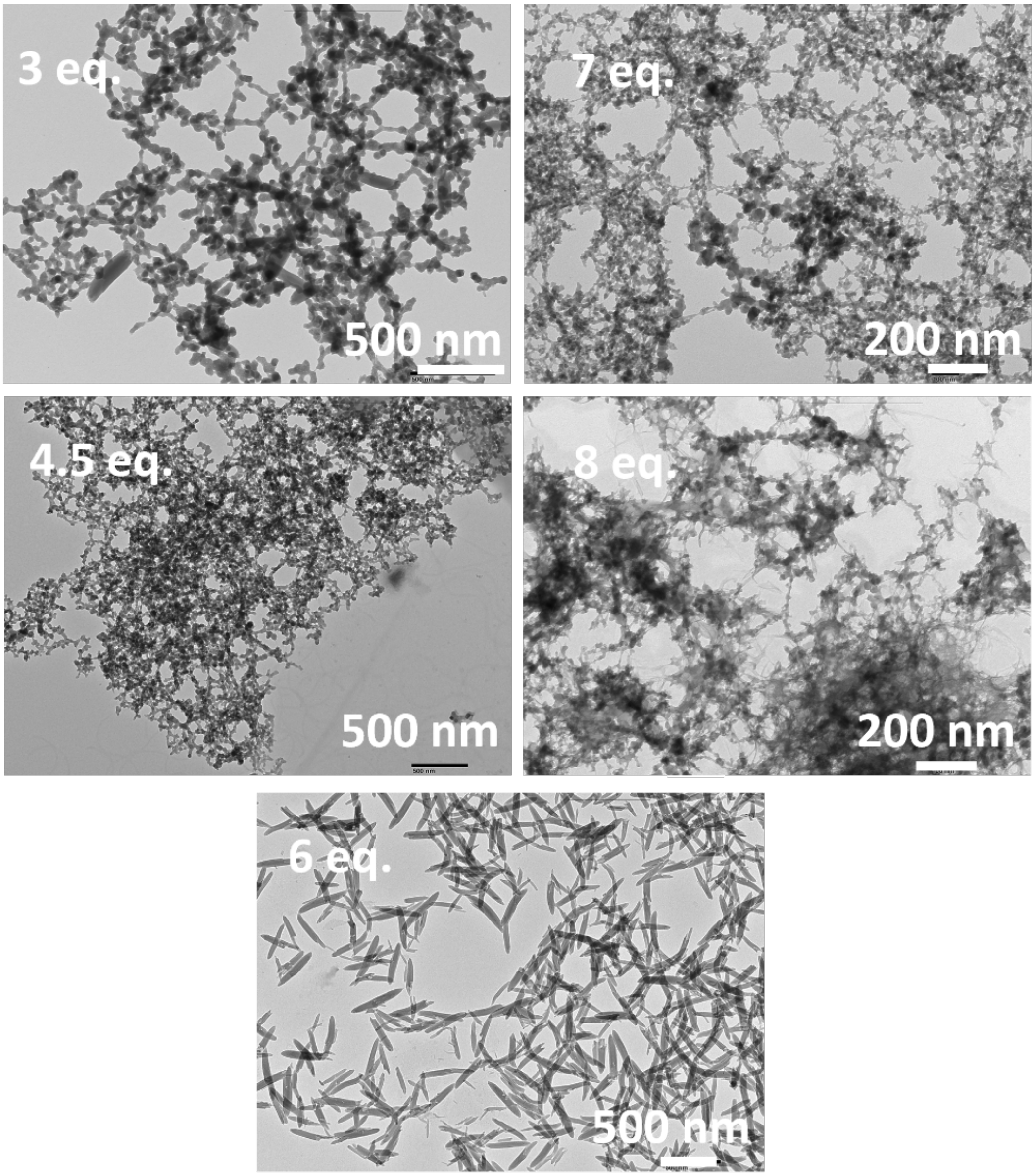

**Figure S3.** TEM images of Al-btc materials obtained with varying NaOH equivalents.

**Synthesis and Characterization of Protein@MIL-110(Al) nanocomposites.**

**Thermogravimetric Analysis (TGA) of the composites**

TGA was used to estimate the protein loading using the **Equation S1**. All curves were normalized at 200 ºC at which temperature physiosorbed water molecules are expected to be removed. From these experimental data and considering the inorganic residues remaining at high temperatures (700 ºC), it is possible to estimate the protein loading by applying the following Equation S1:

$$x\ \% = \frac{residue_{MOF} - residue_{Protein@MOF}}{residue_{MOF}} \cdot 100$$

**Equation S1.** Equation used to calculate protein loading from TGA data. Calculated values are reported in Table 1 in the main text.

**FT-IR Spectroscopy**

FT-IR spectra of Protein@MIL-110(Al) and MIL-110(Al) exhibit the characteristic stretching bands of the btc ligand. For instance, the ν($COO^-$) antisymmetric (1616 $cm^{-1}$) and symmetric (1365 $cm^{-1}$) stretching bands, the ν(C-C) stretching of the benzene ring (1556 $cm^{-1}$) as well as O-H vibration (1437 $cm^{-1}$) are all observed.[4] The most representative bands of encapsulated proteins (e.g. Xylanase) including the ν(C=O) stretching of the amide groups (Amide I, 1629 $cm^{-1}$), C-N stretching coupled to N-H bending (Amide II, 1540 $cm^{-1}$) and N-H in plane bending coupled to C-N stretching (Amide III, 1240 $cm^{-1}$)[5,6] are not detected, as they are covered by the characteristic bands of MIL-110(Al). The bands at 706 and 762 $cm^{-1}$ can be attributed to ν(C-H) of the benzene ring.[7,8] The intense band at ca. 550 $cm^{-1}$ (520 $cm^{-1}$ for the empty MOF) stems from ν(Al-O) stretching bands in different environments (note that there are at least two different aluminium sites in MIL-110(Al) structure).[9,10]

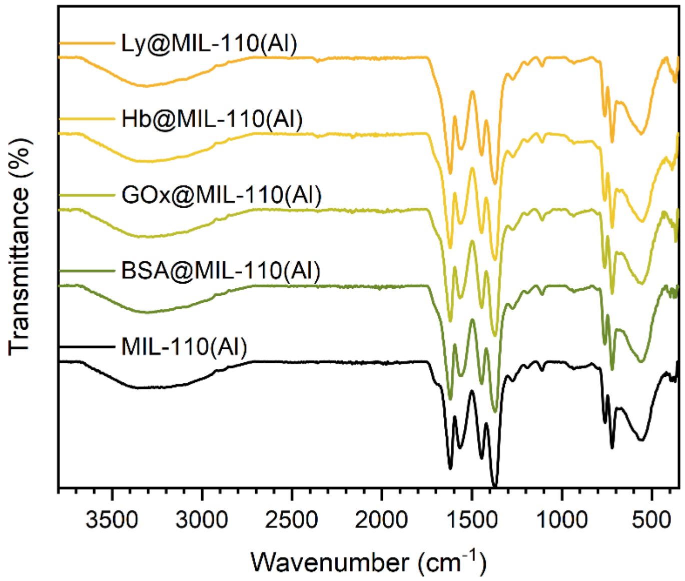


**Figure S4.** FT-IR spectra of protein@MIL-110(Al) composites.

## Transmission Electron Microscopy (TEM)

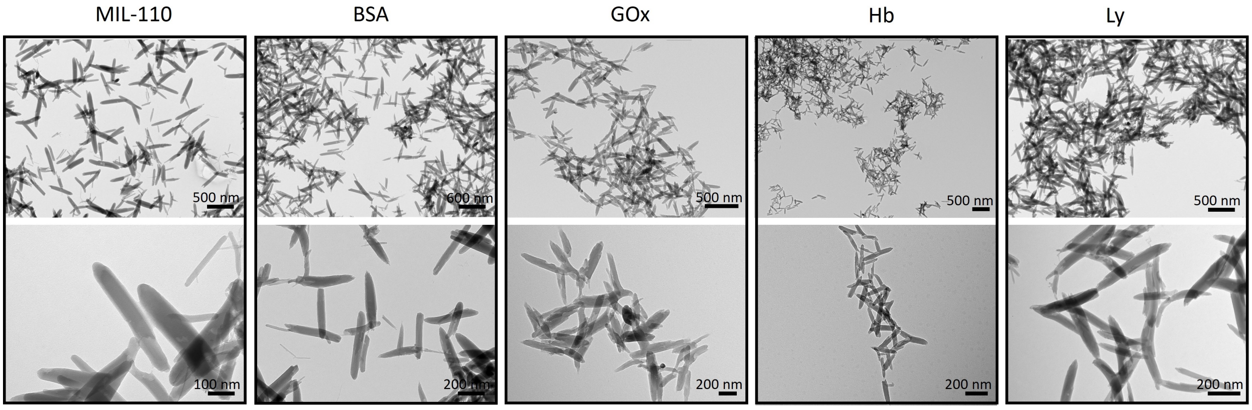


**Figure S5.** TEM images of MIL-110(Al) and protein@MIL-110(Al) composites with BSA, Gox, Hb and Ly.

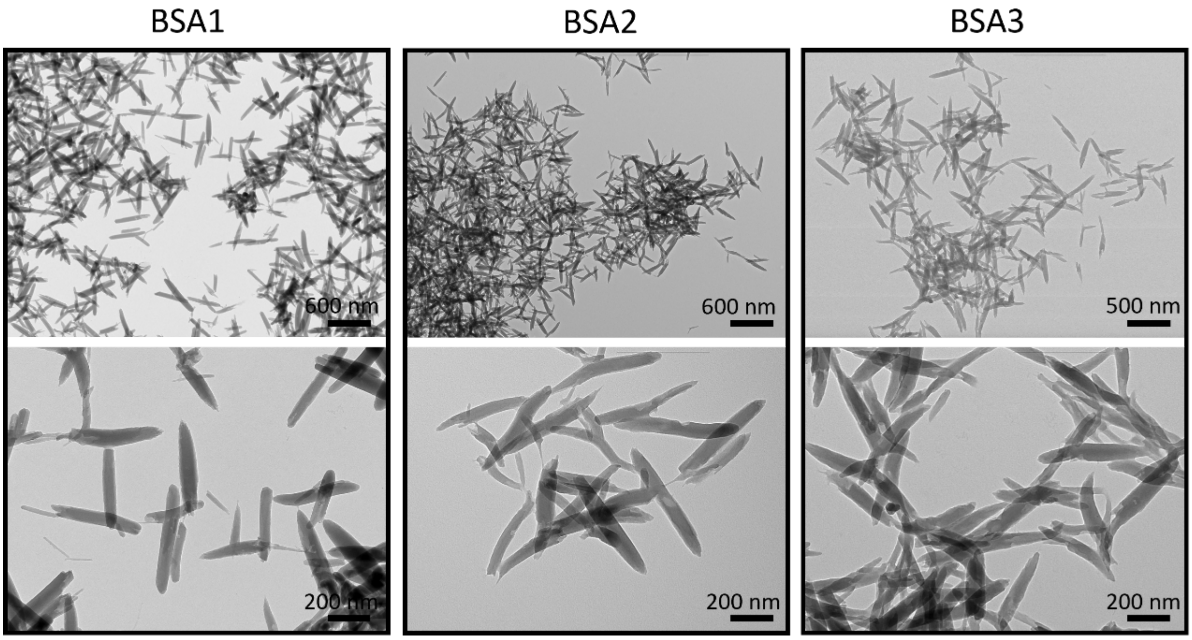


**Figure S6.** TEM images of the three BSA@MIL-110(Al) composites with different loadings.

### Scaling up the synthesis of MIL-110(Al) and composites

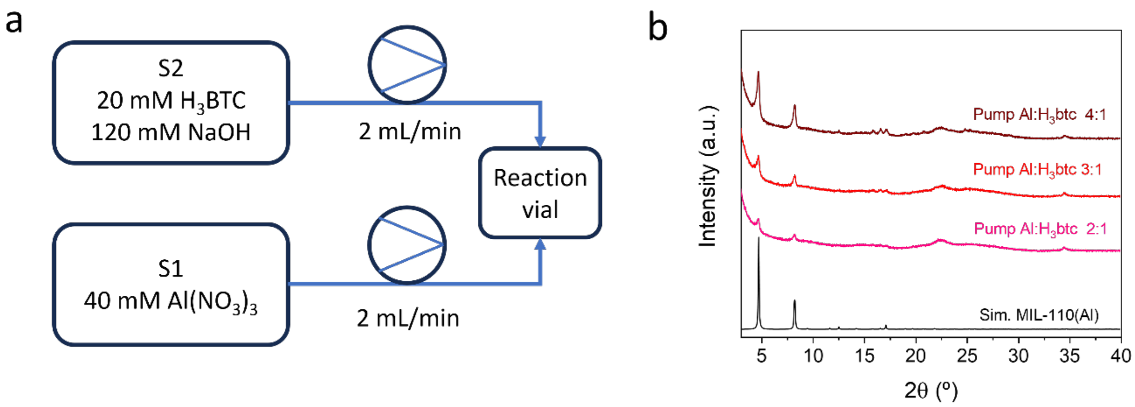


**Figure S7. (a)** Scheme of the simultaneous addition set-up. (b) PXRD of the materials obtained by simultaneous addition with different Al:btc ratios as compared to simulated MIL-110(Al) pattern.

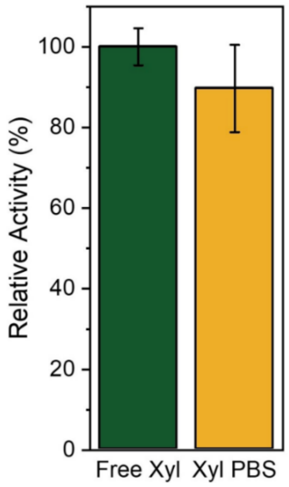


**Figure S8**. Relative activity of xylanase (Xyl PBS) released from MIL-110(Al) upon composite immersion in PBS as compared to the free enzyme.

**Characterization of Xyl@MIL-100(Fe)**

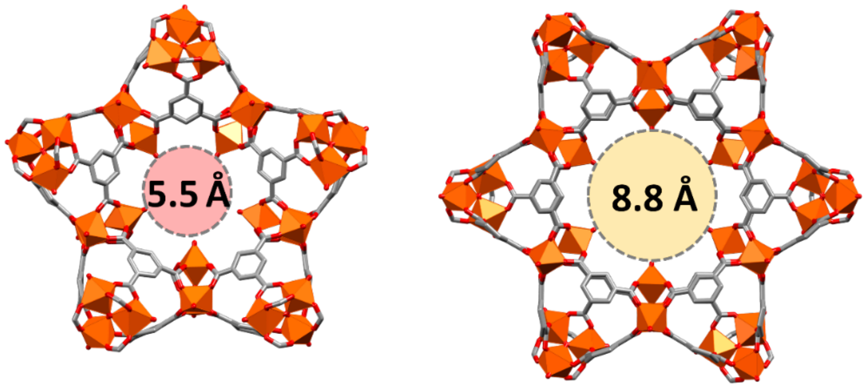


**Figure S9.** Schematic representation of the pore aperture dimensions in the iron(III) trimesate MIL-100(Fe) material.

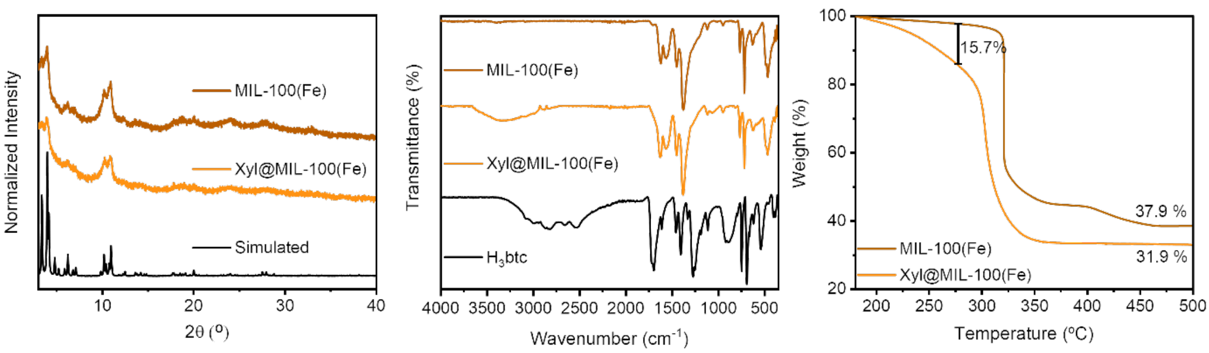


**Figure S10.** PXRD, FT-IR and TGA (from left to right) of Xyl@MIL-100(Fe) and control empty MIL-100(Fe) prepared as previously reported by our group.[1]

**Catalytic Activity of Xylanase@MIL-110(Al) and Xyl@MIL-100(Fe)**

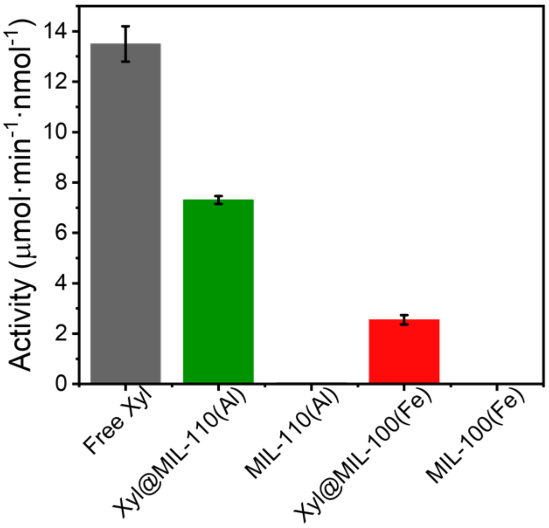


**Figure S11.** Activity of free Xylanase, Xyl@MIL-110(Al) and Xyl@MIL-100(Fe).

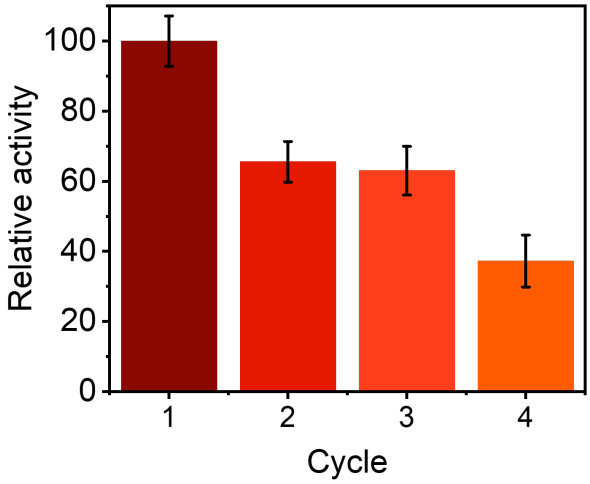


**Figure S12.** Relative activity (to first cycle) of xylanase in Xyl@MIL-100(Fe).

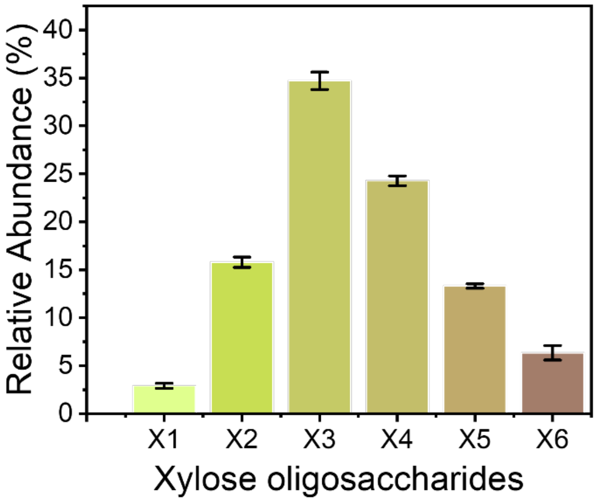


**Figure S13.** Relative abundance of the products catalysed by free Xyn3-CBM9 according to the number of xylose units.

## AUTHOR INFORMATION


### Corresponding Author

**Dr. M. Giménez-Marqués** - Universidad de Valencia - Instituto de Ciencia Molecular, Catedrático José Beltrán Martínez 2, 46980 Paterna, Spain; ORCID: 0000-0002-4931-5711; Email: monica.gimenez-marques@uv.es

### Author Contributions

‡ J.G. and J.C.D. contributed equally to this work.


## REFERENCES


(1) Cases Díaz, J.; Lozano-Torres, B.; Giménez-Marqués, M. Boosting Protein Encapsulation through Lewis-Acid-Mediated Metal–Organic Framework Mineralization: Toward Effective Intracellular Delivery. *Chem. Mater.* **2022**, *34* (17), 7817–7827. https://doi.org/10.1021/acs.chemmater.2c01338.

(2) Talens-Perales, D.; Sánchez-Torres, P.; Marín-Navarro, J.; Polaina, J. In Silico Screening and Experimental Analysis of Family GH11 Xylanases for Applications under Conditions of Alkaline pH and High Temperature. *Biotechnol Biofuels* **2020**, *13* (1), 198. https://doi.org/10.1186/s13068-020-01842-5.

(3) Míguez Amil, S.; Jiménez-Ortega, E.; Ramírez-Escudero, M.; Talens-Perales, D.; Marín-Navarro, J.; Polaina, J.; Sanz-Aparicio, J.; Fernandez-Leiro, R. The Cryo-EM Structure of *Thermotoga Maritima* β-Galactosidase: Quaternary Structure Guides Protein Engineering. *ACS Chem. Biol.* **2020**, *15* (1), 179–188. https://doi.org/10.1021/acschembio.9b00752.

(4) Hadjiivanov, K. I.; Panayotov, D. A.; Mihaylov, M. Y.; Ivanova, E. Z.; Chakarova, K. K.; Andonova, S. M.; Drenchev, N. L. Power of Infrared and Raman Spectroscopies to Characterize Metal-Organic Frameworks and Investigate Their Interaction with Guest Molecules. *Chem. Rev.* **2021**, *121* (3), 1286–1424. https://doi.org/10.1021/acs.chemrev.0c00487.

(5) Singh, B. R.; DeOliveira, D. B.; Fu, F.-N.; Fuller, M. P. Fourier Transform Infrared Analysis of Amide III Bands of Proteins for the Secondary Structure Estimation; Nafie, L. A., Mantsch, H. H., Eds.; Los Angeles, CA, 1993; pp 47–55. https://doi.org/10.1117/12.145242.

(6) Olsztyńska-Janus, S.; Pietruszka, A.; Kiełbowicz, Z.; Czarnecki, M. A. ATR-IR Study of Skin Components: Lipids, Proteins and Water. Part I: Temperature Effect. *Spectrochimica Acta Part A: Molecular and Biomolecular Spectroscopy* **2018**, *188*, 37–49. https://doi.org/10.1016/j.saa.2017.07.001.

(7) Huang, S.; Yang, K.-L.; Liu, X.-F.; Pan, H.; Zhang, H.; Yang, S. MIL-100(Fe)-Catalyzed Efficient Conversion of Hexoses to Lactic Acid. *RSC Adv.* **2017**, *7* (10), 5621–5627. https://doi.org/10.1039/C6RA26469G.

(8) Fukuda, H. Enzymatic Production of Biodiesel. In *Biofuels*; Soetaert, W., Vandamme, E. J., Eds.; Wiley, 2009; pp 129–151. https://doi.org/10.1002/9780470754108.ch8.

(9) Tarte, P. Infra-Red Spectra of Inorganic Aluminates and Characteristic Vibrational Frequencies of $AlO_4$ Tetrahedra and $AlO_6$ Octahedra. *Spectrochimica Acta Part A: Molecular Spectroscopy* **1967**, *23* (7), 2127–2143. https://doi.org/10.1016/0584-8539(67)80100-4.

(10) Rana, S.; Ram, S. X-Ray Diffraction and IR Spectrum for Activated Surface Hydrolysis of Al Metal into AlO(OH)·αH2O Nanocrystals in a New Monoclinic Crystal Structure. *Journal of Solid State Chemistry* **2001**, *157* (1), 40–49. https://doi.org/10.1006/jssc.2000.9034.
